# Characteristics and Age-Related Changes in Orbital Fat Protrusion in East Asians: A Retrospective 3-Dimensional Computed Tomography–Based Study

**DOI:** 10.1093/asjof/ojaf122

**Published:** 2025-09-26

**Authors:** Kiyoko Kato, Itsuko Okuda

## Abstract

**Background:**

East Asians have a high prevalence of orbital fat protrusion (eye bags) from a young age, but underlying changes over time have not been extensively assessed.

**Objectives:**

The aim of the authors of this study is to evaluate age-related changes in orbital fat protrusion and the positional relationship between the eye globe and cheek in East Asians.

**Methods:**

This was a retrospective analysis of adults undergoing head and neck computed tomography at a single center. Various parameters were assessed relative to a pupil-centered reference line, including the most anterior points of: the globe (A); inferior orbital fat pad (F); infraorbital rim (O); and cheek (C). Distances AF, AO, AC, and FO (eye bag prominence) were calculated. The East Asian group was also compared with historical Caucasian data through 1:1 matching.

**Results:**

The East Asian population included 224 participants (age range, 20-79 years). Orbital fat (AF) protruded among young individuals, increasing with age; inferior orbital rim position (AO) remained unchanged over time; the cheek (AC) showed a high prevalence of negative vectors from youth, and increased projection with advancing age; eye bags (FO) were present even in young individuals and became somewhat more pronounced with aging. By comparison, the matched Caucasian cohort (*n* = 22) showed significant decreases in AO and AC and increases in FO with age.

**Conclusions:**

From a young age, East Asians exhibit a high prevalence of orbital fat protrusion. This is largely attributable to inherent structural factors, whereas such protrusion in Caucasians may be primarily because of age-related retrusion of the orbital rim and cheek.

**Level of Evidence:**

4 (Diagnostic) 
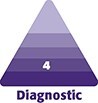

East Asians exhibit a higher prevalence of orbital fat protrusion (eye bags) from a young age, compared with Caucasians.^1,2^ A key proposed reason for this is that East Asians inherently have a more “2-dimensional” facial structure. Specifically, the maxilla is relatively retruded, leading to a negative vectoral relationship between the eye globe and the cheek.^3^

However, few reports have assessed in detail the unique features of orbital fat protrusion, the lower eyelids, and soft tissue characteristics among East Asians. Existing analyses have been limited in scope.^4,5^

The aim of the authors of the present study is to evaluate orbital fat protrusion and the positional relationship between the globe and cheek in East Asian patients, using 3-dimensional (3D) computed tomography (CT) images. In addition, age-related changes were examined, and a comparative statistical analysis was conducted using previously published data from Caucasian individuals.^5^

## METHODS

This was a retrospective observational study approved by the Ethics Committee of Shiba Palace Clinic (Minato-ku, Tokyo, Japan; approval no. 155888_rn-37949). Given the nature of the analyses, patient anonymity was ensured, and informed consent was waived.

### Participant Selection

Medical records were reviewed among adults who underwent head and neck CT scans at the International University of Health and Welfare Mita Hospital (Tokyo, Japan) between July 2014 and December 2022. All relevant patients were included in the present analysis except for those who met any of the following exclusion criteria: severe artifacts in imaging; a history of facial fractures; or significant bony structural changes because of conditions like sinusitis.

### CT Scanning Technique

CT scans were performed using a 320-detector spiral scanner (Aquilion ONE, Canon Medical Systems, Tochigi, Japan) with the following parameters: tube voltage, 120 kVp; tube current, 130 to 180 mA; exposure time, 1 s; and slice thickness, 0.5 mm. CT data were transferred to a workstation (ZioCube, Ziosoft Inc, Tokyo, Japan), where 3D images were reconstructed.

Preset facial reconstruction algorithms were used to create images of the globe, infraorbital rim, orbital fat protrusion, and cheek. Volume rendering was applied based on an edge-detection image-processing system.

### Analysis of CT Images

**Figure 1.**
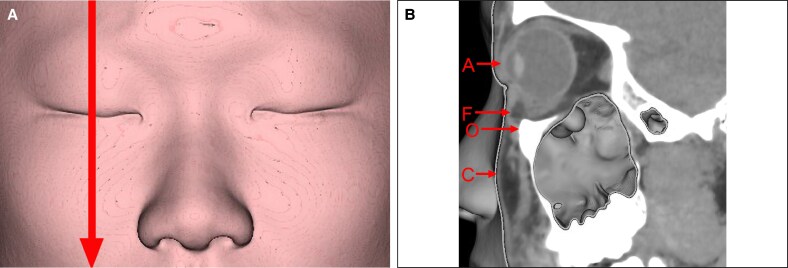
Parameter measurements from computed tomography scans. (A) For each patient, a 3-dimensional reconstruction of the soft tissue was generated, and the mid-pupillary plane on the left side (vertical line) was identified. (B) The orbital view was then aligned with the mid-pupillary plane and visualized from a standard left lateral perspective. The soft tissue reconstruction provided clear visualization of the anterior surface of the globe (Point A), the anterior margin of the inferior orbital fat pad (Point F), the infraorbital rim (Point O), and the most anterior projection of the cheek (Point C).

Measurements were performed using a modified version of the method proposed by Pessa et al.^5^ Various parameters were assessed based on a pupil-centered reference line (Figure 1A). These parameters included the most anterior point of the globe (A); the anterior margin of the inferior orbital fat pad (F); the infraorbital rim (O); and the most anterior projection of the cheek (C) (Figure 1B).

The distances AF, AO, and AC were calculated, and the prominence of the eye bag (FO) was defined based on AF minus AO.

**Figure 2.**
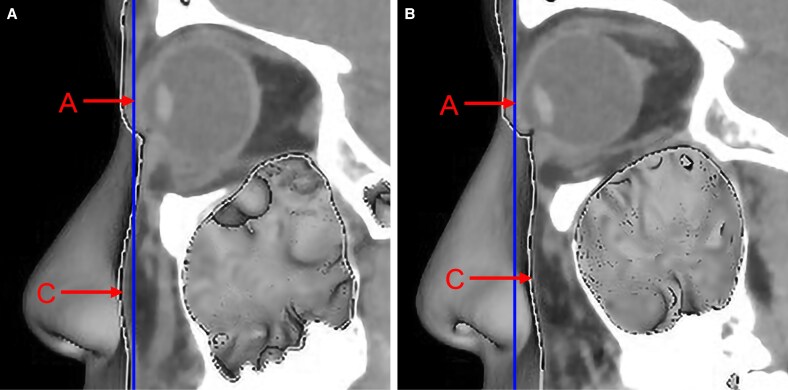
Vector assessment based on the AC value. (A) A positive vector, where the anterior surface of the cheek (Point C) extends anteriorly beyond the globe (Point A). (B) A negative vector, where Point C is positioned posteriorly relative to Point A.

With regard to AC, if the globe was positioned posteriorly relative to the cheek, the vector value was positive (Figure 2A). If they were aligned, the value was zero (neutral vector). If the globe was positioned anteriorly relative to the cheek, the vector value was negative (Figure 2B).^6^

The analyses were performed by 2 observers in consultation with each other—a radiologist with 28 years of experience and an aesthetic plastic surgeon with 17 years of experience. All measurements were taken 3 times, and the average value was recorded.

### Statistical Analyses

Two statistical approaches were taken to evaluating age-related changes. In the first, participants were divided into 3 groups according to their age: Group 1, 20 to 39 years; Group 2, 40 to 59 years; and Group 3, 60 to 79 years. An independent-samples *t*-test was then performed to compare the mean values of the 4 measured parameters (AF, AO, AC, and FO) among the 3 age groups.

In the second age analysis, a linear regression analysis was conducted to evaluate the relationship between age and each the 4 measured parameters (AF, AO, AC, and FO). A Loess regression curve was also created to assess the continuous relationship between age and each parameter.

Data from this East Asian patient group was also compared with previously published results from a Caucasian patient group (from Pessa et al), based on a 1:1 age- and sex-matched analysis.^5^ Welch's *t*-test was used to assess statistical significance in comparisons between young and older individuals in the East Asian and Caucasian groups. Furthermore, a multivariable linear regression analysis with an interaction term for age and ethnicity was performed to evaluate differences in age-related changes between groups.

All statistical analyses were conducted using IBM SPSS Statistics ver. 24.0 (IBM Japan, Ltd, Tokyo, Japan). A *P*-value <.05 was considered to be statistically significant.

## RESULTS

CT scans from 254 eligible individuals were assessed. In all cases, the images were of high quality, with no observable pathological deformities of the bony orbit or surrounding soft tissues. The final analysis included 224 participants. Of these, 103 were male (46%) and 121 were female (54%). The mean age was 46.7 ± 19.5 years (range, 20-79 years).

### Age-Related Changes in East Asians

**Figure 3.**
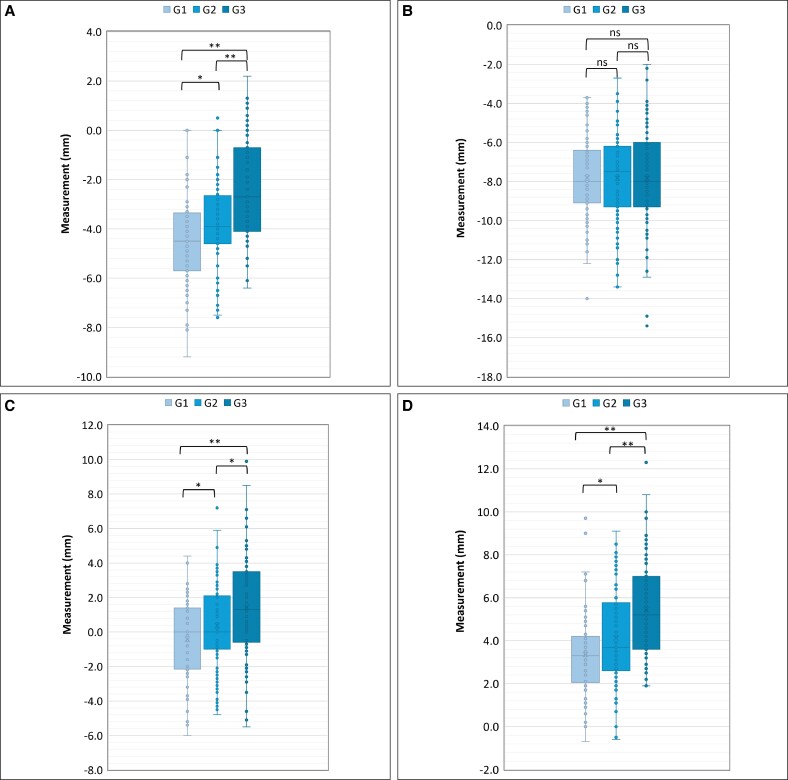
Measured parameters according to age group. Data are provided for participants (individual circles) in Group 1 (20-39 years), Group 2 (40-59 years), and Group 3 (60-70 years). For each age group, the horizontal line represents median, the colored box represents interquartile range (25th-75th percentiles), and the whiskers represent minimum and maximum values, excluding outliers. Data are provided for (A) AF, (B) AO, (C) AC, and (D) FO (eye bag prominence). *P < .01; **P < .001. A most anterior point of the globe; C, most anterior projection of the cheek; F, anterior margin of the inferior orbital fat pad; G, group; O, infraorbital rim; ns, not significant.

Comparisons of AF, AO, AC, and FO in 3 different age groups (Group 1, 20-39 years; Group 2, 40-59 years; Group 3, 60-79 years) are presented in Table 1 and Figure 3. Significant differences between age groups (*P* < .01) were observed for all parameters except AO. In the youngest age group (Group 1), the mean FO value (eye bags) was positive, and the mean AC value was negative, indicating a negative vector (Table 1).

**Table 1.** Measured Parameters Across Different Age Groups

Age group	Participants (n)	Ages (years), mean (SD)	Measurement (mm), mean (SD)			
			AF (mm)	AO (mm)	AC (mm)	FO (mm)
Group 1 (20-39 years)	73	28.9 (4.9)	−4.4 (1.8)	−7.8 (2.2)	−0.4 (2.3)	3.4 (1.9)
Group 2 (40-59 years)	84	49.5 (5.7)	−3.7 (1.7)	−7.8 (2.3)	0.4 (2.5)	4.1 (2.2)
Group 3 (60-79 years)	67	69.0 (5.5)	−2.4 (2.1)	−7.8 (2.7)	1.4 (2.9)	5.4 (2.3)

A, most anterior point of the globe; C, most anterior projection of the cheek; F, anterior margin of the inferior orbital fat pad; O, infraorbital rim; SD, standard deviation.

The results of a linear regression analysis evaluating the relationship between age and each measured parameter are summarized in Table 2. AF, AC, and FO increased significantly with age (*P* < .001), with regression coefficients suggesting increases of 0.051 to 0.057 mm per year in each of these parameters. In contrast, AO showed no significant correlation with age.

**Table 2.** Evaluation of the Relationship Between Age and Measured Parameters in East Asians Using Linear Regression Analysis

Dependent variable	Independent variable	Regression coefficient (95% CI)	*β*	*t*-value	*P*-value
AF	Age	0.057 (0.043, 0.070)	0.460	8.215	<.001
AO	Age	0.005 (−0.013, 0.023)	0.034	0.546	.586
AC	Age	0.051 (0.032, 0.070)	0.313	5.239	<.001
FO (eye bag)	Age	0.052 (0.035, 0.068)	0.369	6.296	<.001

*β* is the standardized regression coefficient (calculated as an indicator of the strength of correlation with age). A, most anterior point of the globe; C, most anterior projection of the cheek; F, anterior margin of the inferior orbital fat pad; O, infraorbital rim.

**Figure 4.**
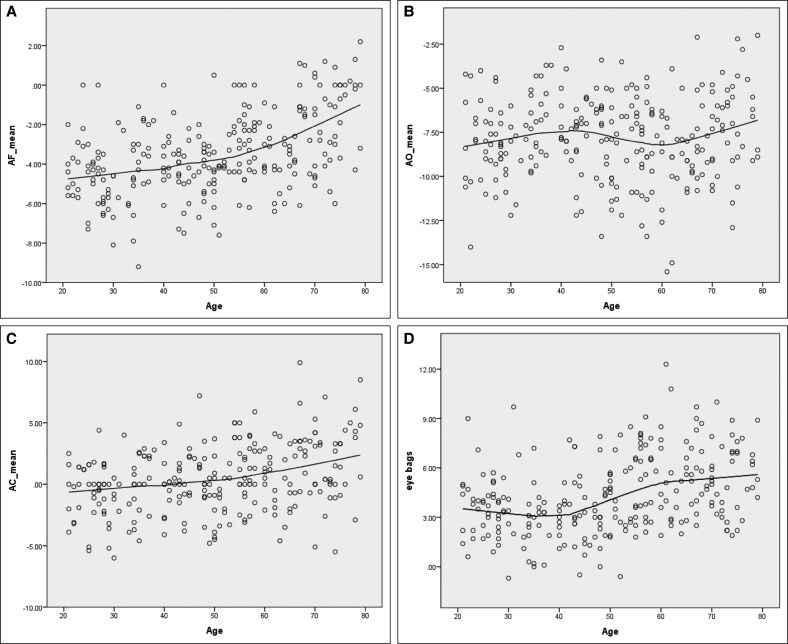
Continuous relationships between age and measured parameters: loess regression curves. Curves are shown for age (A) vs AF, (B) age vs AO, (C) age vs AC, and (D) age vs FO (eye bag prominence). A significant correlation with age was observed for all parameters except AO. A, most anterior point of the globe; C, most anterior projection of the cheek; F, anterior margin of the inferior orbital fat pad; O, infraorbital rim.

Loess regression curves illustrating the continuous relationship between age and each parameter are shown in Figure 4. Orbital fat (AF) showed a tendency to protrude anteriorly from a young age and this increased further with aging (Figure 4A). However, the position of the inferior orbital rim (AO) remained unchanged (Figure 4B). The positional relationship between the globe and the cheek (AC) exhibited a high prevalence of negative vectors from youth, with a trend toward increased value (ie, more anterior projection) with advancing age (Figure 4C). Eye bags (FO) were present even in young individuals and became somewhat more pronounced with aging (Figure 4D).

**Figure 5.**
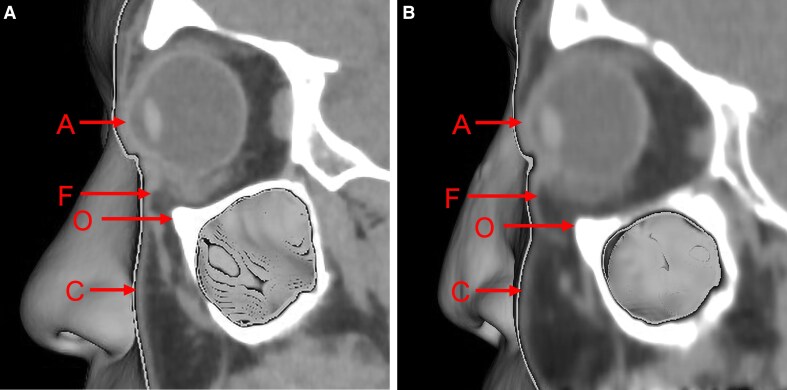
Comparison of measured parameters between typical young and older East Asians. (A) A young individual (a 26-year-old female; AF, −4.2; AO, −9.1; AC, −1.1; FO, +4.3). (B) An older individual (a 68-year-old female; AF, −1.5; AO, −8.5; AC, +2.7; FO, +7.0). A, most anterior point of the globe; C, most anterior projection of the cheek; F, anterior margin of the inferior orbital fat pad; O, infraorbital rim.

Examples of typical age-related periorbital changes in young and older East Asians are shown in Figure 5.

### Matched Comparison With Caucasians

A 1:1 matching between the current East Asian dataset and a historical Caucasian population (from Pessa et al) yielded a total of 22 cases per group (Supplementary Table).^5^ Of these, 10 per group were considered young (aged 18-27 years) and 12 per group were older (aged 43-67 years). The sex ratios were identical, and mean ages were nearly identical between the 2 populations.

Among young patients, East Asians had significantly higher AF and FO (eye bag prominence) values compared with Caucasians (*P* < .001; Table 3). In addition, the frequency of negative AC vectors in young East Asians was 40% compared with 20% in Caucasians. Meanwhile, in older individuals, East Asians exhibited significantly higher AF, AO, and AC values than Caucasians (*P* < .01), but FO values were comparable.

**Table 3.** Comparison of Measured Parameters Between Young and Older East Asians and Caucasians

	East Asians (current study)	Caucasians (1999 study)^5^	Mean difference (SE; 95% CI)	*P*-value
Young individuals	*n* = 10^a^	*n* = 10^a^		
Age, years	22.4 ± 1.9	21.9 ± 2.8		
AF, mm	−4.21 ± 1.27	−7.90 ± 2.09	3.69 (0.77; 2.04, 5.34)	<.001
AO, mm	−8.41 ± 2.92	−8.75 ± 2.42	0.34 (1.20; −2.18, 2.86)	.780
AC, mm	−0.35 ± 2.45	2.05 ± 3.05	−2.40 (1.24; −5.01, 0.21)	.069
FO (eye bag), mm	4.20 ± 2.06	0.85 ± 1.68	3.35 (0.84; 1.58, 5.12)	<.001
Older individuals	*n* = 12^b^	*n* = 12^b^		
Age, years	54.6 ± 7.6	54.5 ± 7.6		
AF, mm	−3.02 ± 2.29	−6.38 ± 2.69	3.36 (1.02; 1.24, 5.48)	.003
AO, mm	−8.03 ± 2.03	−11.21 ± 2.27	3.18 (0.88; 1.36, 5.01)	.002
AC, mm	1.85 ± 2.65	−2.63 ± 3.19	4.48 (1.20; 1.98, 6.97)	.001
FO (eye bag), mm	5.01 ± 2.48	4.83 ± 2.05	0.18 (0.93; −1.75, 2.10)	.852

^a^Six females and 4 males in each group.

^b^Six females and 6 males in each group. Data are mean ± standard deviation unless otherwise stated. Differences between groups were calculated as current study value minus 1999 study value. *P-*values were derived from Welch's *t*-test. A, most anterior point of the globe; C, most anterior projection of the cheek; F, anterior margin of the inferior orbital fat pad; O, infraorbital rim; SE, standard error.

**Figure 6.**
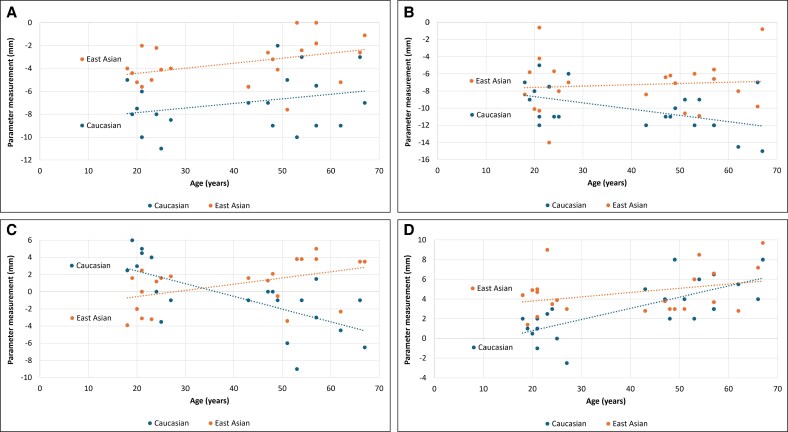
Differences in age-related changes in measured parameters between East Asians and Caucasians. Multivariable linear regression analysis was performed to compare age-related changes in each parameter between East Asians and Caucasians—including (A) AF, (B) AO, (C) AC, and (D) FO. The blue circles and lines represent Caucasians and the orange circles and lines represent East Asians. A, most anterior point of the globe; C, most anterior projection of the cheek; F, anterior margin of the inferior orbital fat pad; O, infraorbital rim.

A multivariable linear regression analysis was performed to compare age-related changes in each parameter between East Asians and Caucasians (Table 4; Figure 6). Relative to East Asians, the Caucasian group showed significant decreases in AO and AC with age, leading to an increased prevalence of negative vectors, whereas FO increased significantly with age.

**Table 4.** Multivariable Linear Regression Model Incorporating the Interaction Term Between Age and Ethnic Group

	Regression coefficient (95% CI)	*t*-value	*P*-value
AF			
Age (Caucasians)	0.040 (−0.014, 0.095)	1.495	.143
Age (East Asians)	0.042 (−0.013, 0.098)	1.539	.132
Group: East Asians vs Caucasians	3.498 (2.17, 4.825)	5.325	<.001
Age × group	0.002 (−0.076, 0.08)	0.046	.963
AO			
Age (Caucasians)	−0.073 (−0.133, −0.013)	−2.457	.018
Age (East Asians)	0.001 (−0.06, 0.062)	0.037	.971
Group: East Asians vs Caucasians	1.901 (0.439, 3.362)	2.628	.012
Age × group	0.074 (−0.011, 0.160)	1.751	.088
AC			
Age (Caucasians)	−0.148 (−0.217, −0.079)	−4.360	<.001
Age (East Asians)	0.069 (0.000, 0.139)	2.016	.051
Group: East Asians vs Caucasians	1.361 (−0.307, 3.030)	1.649	.107
Age × group	0.217 (0.120, 0.315)	4.497	<.001
FO (eye bag)			
Age (Caucasians)	0.113 (0.061, 0.166)	4.395	<.001
Age (East Asians)	0.041 (−0.012, 0.094)	1.568	.125
Group: East Asians vs Caucasians	1.597 (0.329, 2.865)	2.544	.015
Age × group	−0.072 (−0.147, 0.002)	−1.970	.056

This analysis investigated whether there were differences in the slope (ie, age-related changes) for each parameter between East Asians from the present study and Caucasians from the study by Pessa et al.^5^ Multivariable linear regression was conducted with each parameter as the dependent variable. Independent variables included age, group (Caucasians and East Asians), and their interaction (age × group). If a difference in slope existed between groups, the interaction term would be detected as statistically significant. A, most anterior point of the globe; C, most anterior projection of the cheek; F, anterior margin of the inferior orbital fat pad; O, infraorbital rim.

## DISCUSSION

The findings of this study provide insights into the unique characteristics and age-related changes in orbital fat protrusion in an East Asian (Japanese) cohort.

With regard to cheek prominence, East Asians showed a high prevalence of negative vectors between the eye globe and the cheek at a young age—with a tendency toward greater forward projection of the cheek over time. The authors of this study therefore confirm that East Asians often possess an inherently “2-dimensional” facial structure from youth. Although previous research based on Lambros's theory suggested that the cheek undergoes clockwise atrophy with age, our findings indicate the opposite trend.^7^ East Asian skin may resist sagging over time, despite increased fat volume, owing to thicker and denser dermis—attributed to higher melanocyte density, potentially larger fibroblasts, and smaller collagen fiber bundles.^8,9^ However, age-related changes in cheek shape are influenced by multiple factors, including bone, fat, soft tissue, and skin, so further research is warranted.

It was also notable that the positional relationship between the eye globe and the inferior orbital rim showed minimal age-related changes among East Asians. Previous reports have suggested that changes in orbital shape occur primarily in the superomedial and inferolateral regions, with little alteration in the central area.^10,11^ Nonetheless, although studies of Caucasians have reported some resorption even in the central inferior orbital rim.^12,13^ In contrast, East Asian data indicate minimal changes (particularly in women).^14,15^ For example, in a Korean study, the authors suggested that the orbital area remains stable throughout life, which aligns with our findings.^16^ Racial differences in bone density and osteoporosis progression may explain these discrepancies, highlighting the need for further research into facial bone changes across ethnic groups.^17,18^

Our results also showed that orbital fat tended to protrude in East Asians even among young individuals. The study therefore supports the notion that East Asians are predisposed to eye bags from a young age. Despite minimal changes in the inferior orbital rim, eye bag protrusion did increase with aging—although this occurred more slowly than among Caucasians. Age-related changes in East Asians most likely stem from soft tissue alterations, including thinning of the orbital septum, age-related thinning and detachment of the orbicularis oculi muscle, and changes in skin elasticity.^1,19,20^

In contrast, among Caucasians, bony changes contributed significantly to orbital fat prominence, and this appeared to be largely an age-related phenomenon. Over time, Caucasians exhibited significant retrusion of the inferior orbital rim and cheek, whereas East Asians showed less dramatic changes, emphasizing the structural origin of orbital fat prominence in this group.

It has been hypothesized previously that East Asians are predisposed to eye bags because of their inherently 2-dimensional bone structure, with retrusion of the maxilla and inferior orbital rim providing inadequate support for orbital fat.^1-3^ However, our findings contradict this hypothesis. Two possible explanations arise. The first relates to differences in measurement techniques. Indeed, the inferior orbital rim has a complex and variable shape, leading to measurement challenges. The selection of specific measurement points and advancements in CT imaging technology over the 25 years between 1999 and 2024 could have introduced errors. The second possible explanation relates to the smaller orbital volume in East Asians. If there is no difference in globe size between ethnicities, a smaller orbital volume in East Asians might logically result in orbital fat protrusion.^21^ Future studies focusing on racial differences in orbital volume are needed to provide further clarity.

Our results have important clinical implications. Indeed, if the development of eye bags and a negative vector in Caucasians is largely influenced by age-related changes, whereas in East Asians congenital factors play a greater role, treatment strategies may differ substantially. For example, when performing filler treatments in Caucasian patients, an anti-aging approach is also required, incorporating lifting toward the supralateral side and volumization of the cheeks. By contrast, in East Asian patients, correction of the congenital negative vector generally constitutes the primary treatment approach, with only minimal consideration needed for age-related changes.

We must acknowledge the limitations of the present study. In particular, all imaging was performed in the supine position, which may influence lower eyelid appearance. Both eye bag size and cheek height would generally be expected to appear smaller while supine compared with the standing position. Nonetheless, we were still able to identify clear differences compared with the 1999 study of a Caucasian population, which highlights the potential importance of our findings.^5^ Furthermore, other published studies have also evaluated age-related changes in the periorbital region using supine measurements—including soft tissue, orbicularis oculi muscle, and eye bags—suggesting that assessment of such changes under these conditions is feasible.^4,19^ At present, the ability to perform CT imaging in the standing position is very limited, and it is not yet practical to make large-scale measurements. In the future, we hope it will be possible to perform comparative studies between Asian and Caucasian populations in the standing position.

We should also acknowledge that CT technologies have changed significantly over the past 25 years between the historical control study and our analysis. However, to the best of our knowledge, no more recent and potentially comparable study of a Caucasian population has been published. We therefore sought to perform our measurements under conditions that were as consistent as possible with the 1999 report, including the use of cross-sectional images at the pupillary midline and measurements in the supine position.

We also note that our cohort included 224 cases spanning a wide age range, whereas the historical comparator group consisted of only 22 cases. Moreover, although we believe they were all Caucasian, we cannot be certain that some were not from other ethnic groups. Further research is therefore required, involving similar measurements in larger Caucasian cohorts.

## CONCLUSIONS

From a young age, East Asians exhibit a high prevalence of negative vectors between the eye globe and the cheek—and a high prevalence of orbital fat protrusion. Unlike Caucasians, where orbital fat protrusion is primarily a consequence of age-related retrusion of the orbital rim and cheek, eye bags in East Asians appear to be largely attributable to inherent structural factors rather than aging. This has implications for clinical practice and further studies are warranted.

## Supplemental Material

This article contains supplemental material located online at https://doi.org/10.1093/asjof/ojaf122.

## Supplementary Material

ojaf122_Supplementary_Data
