# Supplementary material for: Characteristics and Age-Related Changes in Orbital Fat Protrusion in East Asians: A Retrospective 3-Dimensional Computed Tomography–Based Study
Source: Aesthet Surg J Open Forum. 2025 Sep 26;7:ojaf122. doi: 10.1093/asjof/ojaf122 (PMC12596715; doi:10.1093/asjof/ojaf122)
Supplement: ojaf122_Supplementary_Data [file ojaf122_supplementary_data.docx]

**Supplementary Table 1.** Matched data between the current study and the historical reference study

| **Study** | **Matching ID** | **Age (years)** | **Age group** | **Sex** | **Parameter (mm)** | | | |
| --- | --- | --- | --- | --- | --- | --- | --- | --- |
|  |  |  |  |  | **AF** | **AO** | **AC** | **FO** |
| *Caucasians (1999 study)^5^* | | | | | | | | |
| 1999 | 2 | 18 | Young | Female | –5 | –7 | 2.5 | 2 |
| 1999 | 3 | 20 | Young | Female | –7.5 | –8 | 3 | 0.5 |
| 1999 | 4 | 21 | Young | Female | –10 | –11 | 4.5 | 1 |
| 1999 | 5 | 24 | Young | Female | –8 | –11 | 0 | 3 |
| 1999 | 6 | 25 | Young | Female | –11 | –11 | –3.5 | 0 |
| 1999 | 7 | 27 | Young | Female | –8.5 | –6 | –1 | –2.5 |
| 1999 | 11 | 19 | Young | Male | –8 | –9 | 6 | 1 |
| 1999 | 12 | 21 | Young | Male | –6 | –5 | 5 | –1 |
| 1999 | 13 | 21 | Young | Male | –10 | –12 | 0 | 2 |
| 1999 | 14 | 23 | Young | Male | –5 | –7.5 | 4 | 2.5 |
| 1999 | 16 | 43 | Older | Female | –7 | –12 | –1 | 5 |
| 1999 | 17 | 47 | Older | Female | –7 | –11 | 0 | 4 |
| 1999 | 18 | 48 | Older | Female | –9 | –11 | 0 | 2 |
| 1999 | 19 | 49 | Older | Female | –2 | –10 | –1 | 8 |
| 1999 | 20 | 57 | Older | Female | –5.5 | –12 | 1.5 | 6.5 |
| 1999 | 21 | 62 | Older | Female | –9 | –14.5 | –4.5 | 5.5 |
| 1999 | 22 | 51 | Older | Male | –5 | –9 | –6 | 4 |
| 1999 | 23 | 53 | Older | Male | –10 | –12 | –9 | 2 |
| 1999 | 24 | 54 | Older | Male | –3 | –9 | –1 | 6 |
| 1999 | 25 | 57 | Older | Male | –9 | –12 | –3 | 3 |
| 1999 | 27 | 66 | Older | Male | –3 | –7 | –1 | 4 |
| 1999 | 28 | 67 | Older | Male | –7 | –15 | –6.5 | 8 |
| *East Asians (current study)* | | | | | | | | |
| 2024 | 2 | 21 | Young | Female | –4 | –8.4 | –3.9 | 4.4 |
| 2024 | 3 | 21 | Young | Female | –5.2 | –10.1 | –2 | 4.9 |
| 2024 | 4 | 21 | Young | Female | –2 | –4.2 | 2.5 | 2.2 |
| 2024 | 5 | 24 | Young | Female | –2.2 | –5.7 | 1.2 | 3.5 |
| 2024 | 6 | 25 | Young | Female | –4.1 | –8 | 1.6 | 3.9 |
| 2024 | 7 | 26 | Young | Female | –4 | –7 | 1.8 | 3 |
| 2024 | 11 | 21 | Young | Male | –4.4 | –5.8 | 1.6 | 1.4 |
| 2024 | 12 | 21 | Young | Male | –5.6 | –0.6 | 0 | 5 |
| 2024 | 13 | 22 | Young | Male | –5.6 | –10.3 | –3.1 | 4.7 |
| 2024 | 14 | 22 | Young | Male | –5 | –14 | –3.2 | 9 |
| 2024 | 16 | 45 | Older | Female | –5.6 | –8.4 | 1.6 | 2.8 |
| 2024 | 17 | 47 | Older | Female | –2.6 | –6.4 | 1.3 | 3.8 |
| 2024 | 18 | 48 | Older | Female | –3.2 | –6.2 | 2.1 | 3 |
| 2024 | 19 | 48 | Older | Female | –4.1 | –7.1 | –0.5 | 3 |
| 2024 | 20 | 55 | Older | Female | 0 | –6.6 | 5 | 6.6 |
| 2024 | 21 | 65 | Older | Female | –5.2 | –8 | –2.3 | 2.8 |
| 2024 | 22 | 51 | Older | Male | –7.6 | –10.6 | –3.4 | 3 |
| 2024 | 23 | 54 | Older | Male | 0 | –6 | 3.8 | 6 |
| 2024 | 24 | 54 | Older | Male | –2.4 | –10.9 | 3.8 | 8.5 |
| 2024 | 25 | 55 | Older | Male | –1.8 | –5.5 | 3.8 | 3.7 |
| 2024 | 27 | 66 | Older | Male | –2.6 | –9.8 | 3.5 | 7.2 |
| 2024 | 28 | 67 | Older | Male | –1.1 | –0.8 | 3.5 | 9.7 |

The age calibration (allowable margin of error) was set to ± 3 years. A, most anterior point of the globe; C, most anterior projection of the cheek; F, anterior margin of the inferior orbital fat pad; O, infraorbital rim.
